# The impact of symptom screening on survival among patients with cancer across varying levels of pre‐diagnosis psychiatric care

**DOI:** 10.1002/cam4.4479

**Published:** 2021-12-20

**Authors:** Rinku Sutradhar, Qing Li, Paul Kurdyak, Lisa Barbera

**Affiliations:** ^1^ Division of Biostatistics Dalla Lana School of Public Health University of Toronto Toronto Ontario Canada; ^2^ ICES Toronto Ontario Canada; ^3^ Institute of Health Policy, Management and Evaluation University of Toronto Toronto Ontario Canada; ^4^ Institute for Mental Health Policy Research Center for Addiction and Mental Health Toronto Ontario Canada; ^5^ Department of Oncology Tom Baker Cancer Centre University of Calgary Calgary Alberta Canada

**Keywords:** Edmonton symptom assessment system, multivariable Cox regression, pre‐diagnosis psychiatric care, propensity scores, psycho‐oncology supports, symptom burden

## Abstract

**Background:**

Patients diagnosed with cancer often experience considerable challenges with mental health, and those who had more intense psychiatric care prior to their cancer diagnosis have a higher risk of mortality. As prior research demonstrated a survival benefit among patients screened for symptoms using the Edmonton symptom assessment system (ESAS), this study aims to examine the association between being ESAS‐screened and the risk of mortality across varying intensity levels of pre‐diagnosis psychiatric care utilization.

**Methods:**

We conducted a retrospective matched cohort study using population‐wide administrative databases. All patients diagnosed with cancer in Ontario, Canada, from January 2007 to December 2015 were identified. Propensity score matching was used to pair ESAS‐screened individuals to those not screened. Pairs were also hard matched on a pre‐diagnosis psychiatric care utilization gradient. A multivariable Cox proportional hazards regression model was implemented to estimate the association between ESAS and mortality, for each intensity level of pre‐diagnosis psychiatric care.

**Results:**

The matched cohort consisted of 119,806 patient pairs (ESAS‐screened and not screened), of whom 54,468 (45.5%) pairs had prior outpatient psychiatric care and 2249 (1.8%) pairs had experienced emergency department visits or had been hospitalized for psychiatric care. Overall being exposed to ESAS was significantly associated with a 51% decrease in the hazard of mortality (HR 0.49, 95%CI 0.48–0.50, *p*‐value <0.0001). This association was similar across all levels of prior psychiatric use, however, there was no evidence of a differential impact.

**Conclusion:**

In addition to routinely monitoring symptom severity, including depression, among patients with cancer, it is also important to identify those with preexisting psychiatric comorbidities at the time of diagnosis. This information can be used to ensure that timely and appropriate psycho‐oncology services and psycho‐social supports are offered to help the patient and their family cope during the cancer disease trajectory.

## Graphical abstract

Being screened for symptom burden, including depression, lowers the risk of mortality across varying intensity levels of pre‐diagnosis psychiatric care utilization among patients with cancer. In addition to routinely monitoring symptom severity, it is also important to identify those with pre‐existing psychiatric comorbidities at the time of cancer diagnosis. This information can be used to ensure that timely and appropriate psycho‐oncology services and psycho‐social supports are offered to help the patient and their family cope during the cancer disease trajectory.

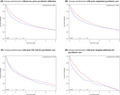

## 1 INTRODUCTION

Patients diagnosed with cancer often experience considerable challenges with mental health.^1^ Compared to healthy populations, cancer patients are at significantly higher risk of depression and death from suicide.^2^, ^3^, ^4^ Mental health challenges following a cancer diagnosis can be even greater among those who experienced mental illness prior to their diagnosis. Recent research demonstrated that patients who sought more intense psychiatric care prior to their cancer diagnosis had a higher risk of death.^5^

Patient reported outcomes (PROs), such as the Edmonton symptom assessment system (ESAS), is a systematic way to identify individuals at high risk of facing issues related to reduced quality of life, and to allow for further assessment and treatment. Routine use of PROs in clinical cancer care can improve longitudinal symptom monitoring, quality of life, and communication among the patient–provider team.^6^, ^7^ The ESAS is a validated and reliable PRO tool for reporting symptom burden across nine domains among patients with cancer.^8^ Symptoms include depression, pain, lack of well‐being, lack of appetite, nausea, anxiety, shortness of breath, tiredness, and drowsiness. The implementation of this tool in Ontario, Canada’s most populous province, initiated in 2007 and has since been adopted in all 14 regional cancer centers and their partner hospitals across the province. With over 5 million symptom records currently captured, Ontario’s cancer system is uniquely positioned to evaluate the impact of symptom screening on longitudinal outcomes at a population level.^9^

There has been growing evidence indicating that routine use of PROs improves survival.^10^, ^11^ It has been recently demonstrated that patients who participated in screening for symptoms using ESAS had a lower risk of mortality compared to those who did not, and this association was strongest within the first year after diagnosis.^12^ As ESAS screens for depression,^13^ this paper aims to expand on prior work by examining the association between symptom screening with ESAS and the risk of mortality across varying intensity levels of pre‐diagnosis psychiatric care utilization. By doing so, we will be able to determine whether the previously observed decrease in mortality associated with ESAS screening is differentially present based on prior psychiatric illness severity.

## 2 METHODS

### 2.1 Study design and population

We conducted a retrospective matched cohort study using population‐wide administrative databases. All patients diagnosed with cancer in Ontario from January 2007 to December 2015 were identified using the Ontario Cancer Registry (OCR), which captures all incident cancers in Ontario.^14^ Patients had to be at least 18 years of age at the time of diagnosis and had to be receiving cancer care from a regional cancer center or partner hospital. Those with a prior history of cancer or multiple cancers were excluded from the study, as were patients without a valid Ontario Health Insurance Plan (OHIP) card.

### 2.2 Data sources

We linked health administrative databases held at the ICES (previously known as the Institute for Clinical Evaluative Sciences). Symptom screening with ESAS was captured using the Symptom Management Reporting Database.^15^ A combination of the Ontario Mental Health Reporting System (OMHRS), Canadian Institute for Health Information's Discharge Abstract Database (CIHI‐DAD) and National Ambulatory Care Reporting System (CIHI‐NACRS), and OHIP were required to determine various levels of psychiatric utilization prior to cancer diagnosis.^16^ Demographic characteristics and date of death were obtained from the Registered Persons Database (RPDB).^17^ The OHIP database was used to retrieve information on physician visits, and visits to emergency rooms were obtained from CIHI‐NACRS. Hospitalizations or same day surgeries were obtained from CIHI‐DAD and CIHI ‐Same Day Surgery database, respectively. Similar to prior work,^12^ the CIHI‐NACRS was used to obtain information on receipt of chemotherapy and radiation therapy within 6 months after diagnosis. The Activity Level Reporting database and New Drug Funding Program database were used to define phase of care along the cancer trajectory. These datasets were linked using unique encoded identifiers and analyzed at ICES.

### 2.3 Main exposure, prior psychiatric utilization, and matching algorithm

The primary exposure was being screened for symptoms with ESAS. Patients diagnosed with cancer who were subsequently screened with ESAS at least once were considered the ESAS‐exposed patients. The index date for the ESAS‐exposed patients was the first ESAS assessment date after their cancer diagnosis. We 1:1 matched each ESAS‐exposed patient to an ESAS‐unexposed patient (diagnosed with cancer but had not been exposed to ESAS). Both hard‐ and propensity score matching were utilized to create this matched cohort. Patients were hard matched on the following criteria: history of psychiatric utilization in the 5 years prior to cancer diagnosis, year of birth (±2 years), date of cancer diagnosis (±1 year), cancer type, and sex. Similar to recent work, psychiatric utilization in the 5 years prior to cancer diagnosis was defined using a gradient with 4‐levels: no psychiatric utilization; outpatient psychiatric care (physician office visits with a diagnosis of depression, schizophrenia, bipolar disorder); emergency department (ED) visit for psychiatric care; and hospital admission for psychiatric care.^4^, ^5^ Each patient was assigned only to the highest level of psychiatric utilization during their prior 5‐year period. This intensity gradient for psychiatric utilization serves as a surrogate for psychiatric disease severity, with the assumption that, on average, individuals who have experienced psychiatric hospitalizations have a greater psychiatric illness severity than those with lower levels of psychiatric utilization intensity.

Logistic regression was used to calculate the propensity of being screened with ESAS. The regression model included: patient characteristics (age, sex, neighborhood income quintile, region of residence), cancer characteristics (type, stage, year of diagnosis), treatments within 6 months of diagnosis (chemotherapy, radiation, and surgery), various measures of comorbid conditions in the 2 years prior to cancer diagnosis (total Charlson comorbidity score,^18^, ^19^ total Aggregated Diagnosis Groups score, and Resource Utilization Bands score from John Hopkins Adjusted Clinical Groups System version 10.0^20^), and number of visits to the emergency department in the 2 years prior to cancer diagnosis. Exposed and unexposed patients were further matched on a caliper width of 0.2 standard deviations of the log odds of the estimated propensity score. It should be noted that 1:1 hard matching of exposed and unexposed individuals based on history of psychiatric utilization allows one to subsequently conduct analyses stratified by the varying levels of utilization, while retaining the paired nature of the data.

Upon completion of matching, a dummy index date was assigned to each unexposed patient such that the gap time (in days) between their diagnosis date and dummy index date was the same as the gap time between the corresponding exposed patient’s diagnosis date and first ESAS date. Patients were followed from their index date until death, diagnosis of a new cancer, 5‐year observation mark, or the end of study at December 31, 2015, whichever came first. Moreover, follow‐up was terminated on unexposed individuals if they had exposure to ESAS after their dummy index date.

### 2.4 Statistical analysis

The distributions of baseline characteristics were compared among patients exposed and unexposed to ESAS, stratified by the four levels of prior psychiatric utilization. Medians and interquartile ranges were used to describe continuous measures, and frequencies and proportions were used to describe categorical measures. Due to the large cohort size, standardized differences (rather than *p*‐values) were used to establish whether covariate distributions were balanced between exposed and unexposed groups; a standardized difference (SD) <0.1 indicated balance.

To illustrate and compare the probability of survival over time for those exposed and unexposed to ESAS, Kaplan–Meier methods, and log‐rank tests were implemented. This was done for the overall matched cohort, and then separately for patients for each of the four levels of prior psychiatric utilization. To determine the association between exposure to ESAS screening and mortality, multivariable Cox proportional hazards regression models were used, and a robust sandwich variance estimation approach was incorporated to account for the matched design. These models were also run on the overall matched cohort, and then separately for matched patients in each of the four levels of prior psychiatric utilization. Since the matching process balanced the distributions of baseline characteristics between the exposed and unexposed groups, the multivariable model only adjusted for the following additional measures: number of visits to a radiation or medical oncologist between cancer diagnosis and index date (included as a fixed covariate measured at index); number of visits to a family physician or radiation/medical oncologist between the index date and end of follow‐up was (included as a counter time‐varying covariate); and experiencing surgery after diagnosis (included as a binary time‐varying covariate that turned “on” once surgery was received). As defined in prior work, phase of care was measured to account for different periods of cancer management across the disease trajectory.^12^ This was incorporated into the Cox model as a 3‐level categorical time‐dependent covariate (initial, continuing, or palliative); the category a patient belonged to depended on the phase of care they were in at that specific point in time. All statistical analyses were done in SAS 14.2 (SAS Institute, Inc.).

## 3 RESULTS

Each pair in our matched cohort consisted of one patient who was exposed to ESAS screening and one patient who was not. The overall matched cohort consisted of 119,806 patient pairs, of whom: 63,089 (52.7%) pairs had no psychiatric utilization in the 5 years prior to cancer diagnosis; 54,468 (45.5%) pairs had prior outpatient psychiatric care; 1480 (1.2%) pairs had experienced prior emergency department visit for psychiatric care; and 769 (0.6%) pairs had been hospitalized for psychiatric care. The distribution of (selected) baseline characteristics among cancer patients with and without ESAS exposure is presented in Table 1. The low standardized differences (<0.1) indicate that the baseline characteristics are well balanced between the ESAS‐exposed and unexposed groups. Fifty three percent of cancer patients who had been hospitalized for psychiatric care prior to their cancer diagnosis were female, and the most common cancer diagnoses among these patients were lung, breast, or prostrate (data not shown).

**TABLE 1:** Distribution of (selected) baseline characteristics among 1:1 matched ESAS‐exposed and unexposed patients, overall

Variable	Value	ESAS screened = No (*N* = 119,806)	ESAS screened = Yes (*N* = 119,806)	SD
Prior psychiatric utilization	None	63,089 (52.7%)	63,089 (52.7%)	0
	Outpatient psychiatric care	54,468 (45.5%)	54,468 (45.5%)	0
	ED visit for psychiatric care	1480 (1.2%)	1480 (1.2%)	0
	Hospital admission for psychiatric care	769 (0.6%)	769 (0.6%)	0
Age	Median (IQR)	65 (57–74)	65 (57–74)	0.01
Cancer type	Brain	1006 (0.8%)	1006 (0.8%)	0
	Breast	17,584 (14.7%)	17,584 (14.7%)	0
	Colorectal	13,406 (11.2%)	13,406 (11.2%)	0
	Gynecological	9048 (7.6%)	9048 (7.6%)	0
	Head and Neck	2955 (2.5%)	2955 (2.5%)	0
	Hematology	15,721 (13.1%)	15,721 (13.1%)	0
	Lung	15,574 (13.0%)	15,574 (13.0%)	0
	Melanoma	4230 (3.5%)	4230 (3.5%)	0
	Non‐melanoma	237 (0.2%)	237 (0.2%)	0
	Other	1190 (1.0%)	1190 (1.0%)	0
	Other Gastrointestinal	8691 (7.3%)	8691 (7.3%)	0
	Other Genitourinary	5245 (4.4%)	5245 (4.4%)	0
	Prostate	22,199 (18.5%)	22,199 (18.5%)	0
	Thyroid	2101 (1.8%)	2101 (1.8%)	0
	Unknown primary	619 (0.5%)	619 (0.5%)	0
Sex	Female	56,673 (47.3%)	56,673 (47.3%)	0
	Male	63,133 (52.7%)	63,133 (52.7%)	0
Stage	0	352 (0.3%)	305 (0.3%)	0.01
	1	28,805 (24.0%)	25,630 (21.4%)	0.06
	2	28,264 (23.6%)	27,129 (22.6%)	0.02
	3	14,858 (12.4%)	15,614 (13.0%)	0.02
	4	14,704 (12.3%)	18,859 (15.7%)	0.1
	Unknown	32,823 (27.4%)	32,269 (26.9%)	0.01
Income quintile	1	21,165 (17.7%)	21,513 (18.0%)	0.01
	2	23,904 (20.0%)	23,760 (19.8%)	0
	3	23,653 (19.7%)	23,748 (19.8%)	0
	4	25,333 (21.1%)	25,183 (21.0%)	0
	5 (wealthiest)	25,751 (21.5%)	25,602 (21.4%)	0
Admissions in 2 years prior to diagnosis	0	100,323 (83.7%)	100,012 (83.5%)	0.01
	1	14,260 (11.9%)	14,536 (12.1%)	0.01
	2	3465 (2.9%)	3513 (2.9%)	0
	3+	1758 (1.5%)	1745 (1.5%)	0
ED visits in 2 years prior to diagnosis	0	61,417 (51.3%)	61,184 (51.1%)	0
	1	28,720 (24.0%)	27,891 (23.3%)	0.02
	2	13,305 (11.1%)	13,463 (11.2%)	0
	3+	16,364 (13.7%)	17,268 (14.4%)	0.02
Charlson comorbidity Score	0	105,991 (88.5%)	105,609 (88.2%)	0.01
	1	6920 (5.8%)	6860 (5.7%)	0
	2	3807 (3.2%)	4051 (3.4%)	0.01
	3+	3088 (2.6%)	3286 (2.7%)	0.01
Aggregated diagnosis group score	0–9	86,340 (72.1%)	86,252 (72.0%)	0
	10+	33,466 (27.9%)	33,554 (28.0%)	0
Resource utilization band	0 (lowest)	806 (0.7%)	663 (0.6%)	0.02
	1	751 (0.6%)	776 (0.6%)	0
	2	4616 (3.9%)	4490 (3.7%)	0.01
	3	59,056 (49.3%)	59,012 (49.3%)	0
	4	32,595 (27.2%)	32,740 (27.3%)	0
	5 (highest)	21,982 (18.3%)	22,125 (18.5%)	0
Received surgery	No	55,353 (46.2%)	59,545 (49.7%)	0.07
	Yes	64,453 (53.8%)	60,261 (50.3%)	0.07
Received chemotherapy	No	90,991 (75.9%)	89,494 (74.7%)	0.03
	Yes	28,815 (24.1%)	30,312 (25.3%)	0.03
Received radiation	No	94,390 (78.8%)	92,909 (77.5%)	0.03
	Yes	25,416 (21.2%)	26,897 (22.5%)	0.03

Note

This table reflects the distributions of patient characteristics at *index*.

**FIGURE 1:**
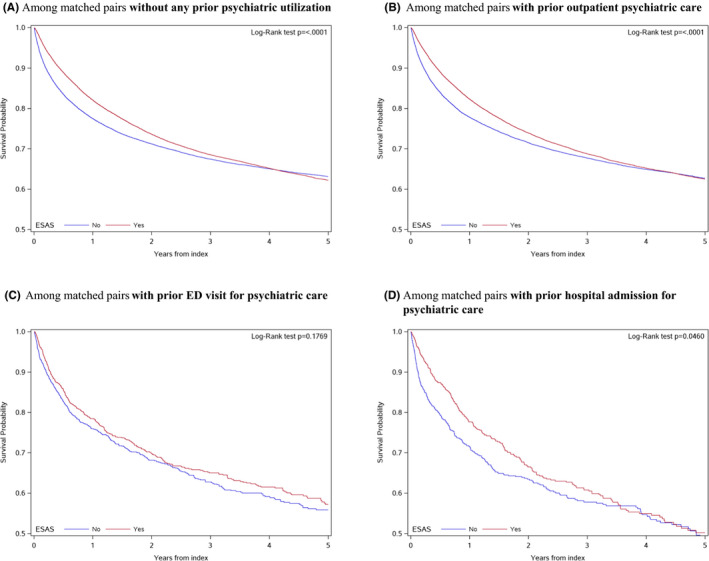
Kaplan–Meier plots illustrating the probability of survival over time for those screened and not screened with ESAS, stratified by each level of prior psychiatric utilization severity: (A) Among matched pairs without any prior psychiatric utilization, (B) Among matched pairs with prior outpatient psychiatric care, (C) Among matched pairs with prior ED visit for psychiatric care, (D) Among matched pairs with prior hospital admission for psychiatric care

Figure 1 illustrates the estimated survival probability over time comparing those exposed versus not exposed to ESAS screening, stratified by severity of prior psychiatric utilization. The survival probability is consistently higher for patients who participated in ESAS screening compared to matched patients who did not. This trend in ESAS impact on survival was maintained over time among cancer patients, irrespective of the level of pre‐diagnosis psychiatric care utilization. Moreover, the risk of mortality was higher for patients who had more intense prior psychiatric use.

Table 2 presents the association between ESAS screening and mortality estimated from the multivariable Cox proportional hazards regression model, among the overall matched cohort. Being exposed to ESAS was significantly associated with a 51% decrease in the hazard of mortality (HR 0.49, 95%CI 0.48–0.50, *p*‐value <0.0001). There was a gradient in the association between prior psychiatric care utilization and mortality. Compared to patients without any prior psychiatric care utilization, those with the highest level of psychiatric care utilization prior to diagnosis had a 25% higher hazard of mortality (HR 1.25, 95%CI 1.14–1.38, *p*‐value <0.0001), whereas those with prior outpatient psychiatric care only did not significantly differ in their hazard of mortality (HR 0.98, 95%CI 0.97–1.003, *p*‐value <0.0001). Compared to patients in the continuing phase of care, those in the palliative phase of care had a 29‐fold higher hazard of death (HR 29.43, 95%CI 28.45–30.45, *p*‐value <0.0001). Once surgery was received, the hazard of mortality notably decreased by 43% (HR 0.57, 95% CI 0.55–0.58, *p*‐value <0.0001).

**TABLE 2:** Estimated adjusted hazard ratio for the association between ESAS exposure and mortality, overall

Comparison		Hazard Ratio	LCL	UCL
ESAS screened	Yes vs. No	0.49	0.48	0.50
Prior psychiatric utilization	Level 3 vs. Level 0	1.25	1.14	1.38
	Level 2 vs. Level 0	1.13	1.05	1.22
	Level 1 vs. Level 0	0.98	0.97	1.003
Phase of care	Initial vs. Continuing	1.37	1.31	1.44
	Palliative vs. Continuing	29.43	28.45	30.45
Received surgery	Yes vs. No	0.57	0.55	0.58
Each additional MedOnc/RadOnc visit from dx to index		0.965	0.963	0.967
Each additional Family/MedOnc/RadOnc visit after index		1.025	1.022	1.027

Note

Level 0 represents lowest level of prior psychiatric utilization.

Table 3 provides the association between ESAS screening and mortality, where matched pairs were stratified by the four levels of pre‐diagnosis psychiatric care utilization. Similar associations were found throughout, both in direction and magnitude of hazard ratios. Being exposed to ESAS significantly decreased the hazard of mortality across all levels of prior psychiatric use, however, there was no evidence of a differential impact.

**TABLE 3:** Estimated adjusted hazard ratio for the association between ESAS exposure and mortality, stratified by prior history of psychiatric care utilization

		Among matched pairs without any prior psychiatric utilization			Among matched pairs with prior outpatient psychiatric care			Among matched pairs with prior ED visit for psychiatric care			Among matched pairs with prior hospital admission for psychiatric care		
Comparison		Hazard Ratio	LCL	UCL	Hazard Ratio	LCL	UCL	Hazard Ratio	LCL	UCL	Hazard Ratio	LCL	UCL
ESAS screened	Yes vs. No	0.48	0.47	0.49	0.49	0.48	0.50	0.54	0.47	0.62	0.55	0.45	0.66
Phase of care	Initial vs. Continuing	1.44	1.34	1.54	1.33	1.24	1.43	1.15	0.81	1.63	0.95	0.63	1.43
	Palliative vs. Continuing	32.13	30.54	33.80	27.75	26.43	29.13	18.85	14.85	23.91	11.19	8.51	14.72
Received surgery	Yes vs. No	0.60	0.59	0.62	0.53	0.52	0.54	0.47	0.40	0.56	0.51	0.41	0.63
Each additional MedOnc/RadOnc visit from dx to index		0.965	0.961	0.968	0.965	0.962	0.969	0.946	0.925	0.967	0.967	0.942	0.994
Each additional Family/MedOnc/RadOnc visit after index		1.028	1.022	1.034	1.023	1.020	1.026	1.008	1.000	1.016	1.002	0.990	1.014

## 4 DISCUSSION

This population‐based matched cohort study among patients diagnosed with cancer examined the association between symptom screening with ESAS and the risk of mortality across varying intensity levels of pre‐diagnosis psychiatric care utilization. We found a strong relationship between ESAS screening and survival. Use of ESAS was associated with a decreased risk in mortality, and this significant reduction was consistent across the intensity gradient for pre‐diagnosis psychiatric care utilization. Although patients with higher levels of prior psychiatric use had a greater risk of mortality, the impact of ESAS on mortality was similar across each level.

Our results are consistent with previous studies assessing prior psychiatric comorbidities and mortality among patients with cancer. Klassen et al. demonstrated that higher intensities of pre‐cancer diagnosis psychiatric utilization were associated with poorer survival.^4^, ^5^ They speculated that the association may be due to several reasons such as: major depression and stress causing biological changes that hinder the body’s immune surveillance to detect cancer at an early stage; and mental illness resulting in a lack of adherence to cancer care follow‐up schedules. Recommendations were made to flag individuals at diagnosis who had high levels of prior psychiatric care so that increased psycho‐social supports could to be offered throughout their cancer trajectory.

We demonstrated that screening for severity of symptoms, including depression, using ESAS was associated with an improvement in survival among all patients, including those with higher intensities of pre‐diagnosis psychiatric care utilization. Randomized studies have also indicated that routine symptom monitoring in cancer patients with solid tumors on chemotherapy was associated with a decreased mortality risk compared to those who were not being routinely monitored for their symptoms.^10^ Another study reported improved survival with weekly symptom monitoring at home among patients with lung cancer, compared to those who were monitored less frequently during cancer clinic visits.^11^ This benefit in survival may be due to symptom screening resulting in earlier identification of symptoms so that a timely and appropriate cancer care management plan can be formulated for the patient. Continued monitoring of symptoms may also ensure that the patient’s care management plan is adjusted accordingly, which in turn may allow patients to continue treatments such as chemotherapy for longer, if needed.^12^

Like other studies, we recommend PROs such as ESAS be administered near the time of a patient’s cancer diagnosis and continue to be used routinely thereafter for screening and management of symptoms, including depression. The impact of ESAS screening on survival was shown to be strongest during the phases of initial / active treatment and palliative care,^12^ so heightened attention to the use of PROs should be considered in these periods. Near the time of diagnosis, it is also important to identify patients with preexisting psychiatric comorbidities so that appropriate psycho‐oncology services and psycho‐social supports can be offered to help the patient and their family cope during the disease trajectory. These recommendations can provide a deeper understanding of a patient’s risk and needs so that a more holistic cancer care management plan, including access to mental health counselling or music therapy, can be developed.

Our study has numerous strengths. We used population‐based health administrative data to create a matched cohort (ESA‐screened vs. not) of nearly 120,000 pairs of patients, accounting for varying intensities of psychiatric care prior to the cancer diagnosis. To minimize confounding both hard‐ and propensity score matching techniques were implemented, resulting in the distributions of patient characteristics to be well balanced at baseline. Changes in measures such as receipt of surgery occurring after diagnosis were appropriately accounted for as time‐varying characteristics. The results from this study are likely generalizable to other populations of cancer patients receiving universal healthcare, and add to the literature on real world evidence surrounding the impact of routine use of PROs in clinical cancer care. However, this study also has several limitations. We were not able to measure other clinical prognostic variables such as performance or functional status. Information on specific chemotherapy regimens and medications for dealing with symptoms such as depression were not available. It is also unclear if our findings could be generalized to cancer populations with access to private healthcare.

To our knowledge, this is the first study to assess the association between symptom screening and mortality across varying levels of pre‐cancer diagnosis psychiatric care utilization. Improvements in survival for those screened with ESAS, seen across the intensity gradient for psychiatric use, highlight the importance of routinely using PROs in clinical cancer. This study also emphasizes the need for patients with cancer to be cared for by a diverse team of providers who can offer supportive cancer care to reduce stress and improve mental well‐being.

## CONFLICT OF INTEREST

The authors declare that they have no conflict of interest.

## AUTHOR CONTRIBUTIONS

All authors had roles in design and conduct of the study. RS and QL planned and executed the statistical analyses. All authors had roles in the interpretation of the results, as well as preparation and approval of the Article.

## ETHICAL STANDARDS

This study involved secondary data analyses only and was thus exempt from requiring REB approval because ICES is a designated “45.1 entity” under the Personal Health Information Protection Act (PHIPA) enabling the use of personal health information.

## Data Availability

Research data are not shared.
